# Interleukin-1β depresses neuronal activity in the rat olfactory bulb even during odor stimulation

**DOI:** 10.1371/journal.pone.0332592

**Published:** 2025-09-18

**Authors:** Adriana Jiménez, Josué Denichi Sánchez-Hernández, Viani Maya-López, Enrique Estudillo, Juan Carlos González-Orozco, Joaquín Manjarrez-Marmolejo, Nieves Herrera-Mundo, Mara A. Guzmán-Ruiz, Rosalinda Guevara-Guzmán

**Affiliations:** 1 División de Investigación, Hospital Juárez de México, Gustavo A. Madero, Ciudad de México, México; 2 Laboratorio de Fisiología de la Formación Reticular, Instituto Nacional de Neurología y Neurocirugía Manuel Velasco Suárez, Tlalpan, Ciudad de México, México; 3 Departamento de Fisiología, Facultad de Medicina, Universidad Nacional Autónoma de México, Coyoacán, Ciudad de México, México; 4 Laboratorio de Reprogramación Celular, Instituto Nacional de Neurología y Neurocirugía Manuel Velasco Suárez, Tlalpan, Ciudad de México, México; 5 Instituto de Fisiología Celular, Universidad Nacional Autónoma de México, Coyoacán, Ciudad de México, México; 6 Departamento de Biología Celular y Fisiología, Instituto de Investigaciones Biomédicas, Universidad Nacional Autónoma de México, Coyoacán, Ciudad de México, México; Eötvös Loránd Research Network Biological Research Centre, HUNGARY

## Abstract

The olfactory system is exposed to external and internal harmful agents that may impair the communication between the olfactory sensory neurons and olfactory brain areas. Inflammatory molecules increase in the olfactory system in response to infections and chronic systemic diseases. Interleukin-1β (IL-1β) is a cytokine produced in many inflammatory processes. In previous studies, we observed that IL-1β increased in the olfactory bulb (OB) of diabetic rats, which also presented olfactory dysfunction. This study aimed to determine whether IL-1β could be responsible for the olfactory impairment. To address this question, IL-1β and its antagonist IL-1Ra were microinjected in the OB of rats to evaluate the electrophysiological activity in the OB and entorhinal cortex (EC) by recording the local field potentials (LFPs) in resting conditions and during olfactory stimulation. RNA-seq analysis from NCBI databases demonstrated the expression of IL-1β receptor 1 (IL1-R1) in the OB from rats and mice. Interestingly, IL-1β reduced total spectral power in the OB and increased total signal frequency and gamma power in both OB and EC. Moreover, IL-1β reduced the amplitude and increased the latency of the olfactory evoked potentials (OEPs) after OB stimulation with amyl acetate. IL-1Ra microinjection before IL-1β rescued amplitude and latency of OEPs, but only partially reverted the effects of IL-1β in total spectral power and relative gamma power. In addition, IL-1Ra changed the electrophysiological activity of OB and EC; however, its effect was lower than that of IL-1β. These results suggest that IL-1β may induce olfactory dysfunction by suppressing neuronal activity in the OB and EC. Furthermore, IL-1β may also have a physiological role in the olfactory system since IL-1Ra can modify the electrical activity in these brain areas.

## Introduction

Olfaction is a chemical sense that allows us to detect and interpret environmental information related to feeding and social behaviors. Remarkably, it also impacts our cognitive abilities and mood state [1]. The olfactory system begins in the nasal cavity, where the olfactory epithelium harbors olfactory sensory neurons (OSN), which interact with odorant molecules and send the olfactory information to the brain. OSN axons cross the cribriform plate and make synapses with the mitral/tufted cells (MTC) in the olfactory bulb (OB), which in turn communicate with secondary olfactory structures that include the piriform and entorhinal cortices [2].

Olfactory dysfunction is an early symptom of different neurodegenerative ailments like Parkinson´s and Alzheimer´s diseases [1,3] and is present in people with chronic degenerative conditions that increase the risk of brain disturbances, such as diabetes [4]. Olfactory impairment is also described in animal models of diabetes [5–7]. In a previous study, we found increased expression of the proinflammatory cytokine interleukin 1β (IL-1β) in the OB of diabetic rats with olfactory dysfunction [8]. Similarly, sustained inflammation mediated by the tumor necrosis factor-α (TNF-α) impairs olfaction by inducing OSN death and affecting the electrophysiological response in the olfactory epithelium of mice [9]. Furthermore, distant inflammation caused by spinal cord injury in mice increased the expression of IL-6 and IL-1β in the OB [10]. Altogether, this information suggests that neuroinflammation plays a key role in the development of olfactory dysfunction.

Inflammatory cytokines are produced in the central nervous system (CNS) mainly by glial cells, showing increased levels after CNS injury or infection, but some cytokines are constitutively produced under physiological conditions [11]. IL-1β is one of the most studied cytokines in inflammation, and glial cells and neurons in the CNS produce it at low levels in physiological conditions, as well as its receptor IL-1R1. In the brain, IL-1β signaling modulates hypothalamic functions such as the regulation of body temperature, sleep, and hippocampal long-term potentiation (LTP) [12]. IL-1β levels increase with brain injury, and its binding to IL-1R1 on the cell surface activates the transcription factor NF-kB, which in turn induces the expression of proinflammatory factors and the recruitment of immune cells to promote neuroinflammation [13].

Overexpression of IL-1β and other proinflammatory cytokines in the olfactory system is mainly described in sinonasal diseases and animal models of inflammation induced by the administration of inflammatory agents such as lipopolysaccharide (LPS), in which atrophy of olfactory structures is associated with olfactory dysfunction [14]. Few works have evaluated the mechanisms implicated in the olfactory damage mediated by IL-1β. One study found a positive correlation between the expression of IL-1β induced by carbon nanoparticles and lipoteichoic acid with the extracellular levels of glutamate and glycine in the OB [15]. Other work demonstrated that the inflammatory response induced by intranasal administration of LPS in mice stimulates the overexpression and aggregation of α-synuclein in the OB in an IL-1β-dependent manner, since LPS did not induce α-synuclein aggregation in IL-1R1 knockout mice [16]. These findings highlight the relevance of IL-1β signaling in the neurodegenerative process of the olfactory system. However, studies focused on deciphering the mechanisms through which IL-1β impairs molecular, cellular, and functional aspects of the olfactory system are needed. In this work, we evaluated the effect of microinjected IL-1β and its antagonist IL-1Ra on the neuronal activity of the OB and entorhinal cortex (EC).

## Materials and methods

### Reagents

Microinjections were performed with carrier-free recombinant human IL-1β (R&D Systems, 201-LB-100/CF) and recombinant human IL-1Ra (Merck, SRP3327−100UG) dissolved in sterile phosphate buffer solution (PBS) at 50 and 25 ng/μl, respectively. Vehicle (PBS) was used for the control group, and 10 ng of IL-1β or IL-1Ra for treatments. The dose of IL-1β and IL-1Ra was established based on results from studies that investigated the effect of these cytokines on the brain [17–22]. The effect of 10 ng of IL-1β and IL-1Ra was addressed in a preliminary recording of the local field potentials (LFPs) in the OB. A single dose of 10 ng of IL-1β decreased LFPs amplitude in the OB immediately after its microinjection, reaching a maximum effect at approximately 10 min and remaining constant for at least 45 min; similar results were observed with a single dose of 10 ng of IL-1Ra (S1 Fig). Based on these preliminary results, LFPs and olfactory evoked response to amyl acetate were evaluated 10 minutes after IL-1β or IL-1Ra microinjection.

### Animals

Adult male Wistar rats (280–320 g) were provided by the Unidad Académica Bioterio (UAB) of the Facultad de Medicina at Universidad Nacional Autónoma de México. Rats were maintained in a 12 h dark/light cycle (dark 8 am-8 pm) with regulated temperature and humidity, and free access to food (5001 PMI Nutrition International Inc.) and water. Experimental procedures were carried out minimizing animal suffering following the Mexican Official Norm NOM-062-ZOO-1999 and the requirements of the World Medical Association Declaration of Helsinki. The study was approved by the Comité Interno para el Cuidado y Uso de Animales de Laboratorio (CICUAL) and the Ethical Committee of the Facultad de Medicina at Universidad Nacional Autónoma de México (FM/DI/043/2022) and Hospital Juárez de México (HJM 044/22-I).

### Recording electrode and cannula implantation

Rats were anesthetized with ketamine/xylazine (90/10 mg/kg) and placed in a rodent stereotaxic frame (David Kopf, USA) to expose the skull and introduce an unilateral bipolar recording electrode (teflon-covered stainless steel wire, 50 µm diameter) attached to a guide cannula (stainless steel, 23 gauge) in the right OB using the coordinates 6.9 mm anteroposterior, 1.5 mm lateral, and 4.0 mm vertical. Another bipolar recording electrode was placed in the EC using the coordinates −5.4 mm anteroposterior, 6.8 mm lateral, and 8.2 mm vertical [23]. The reference electrode was placed in the occipital bone. Electrodes and cannula were fixed to the skull with dental acrylic. Electrode impedance was maintained below 4 MΩ.

### Local field potential recording

**Fig 1:**
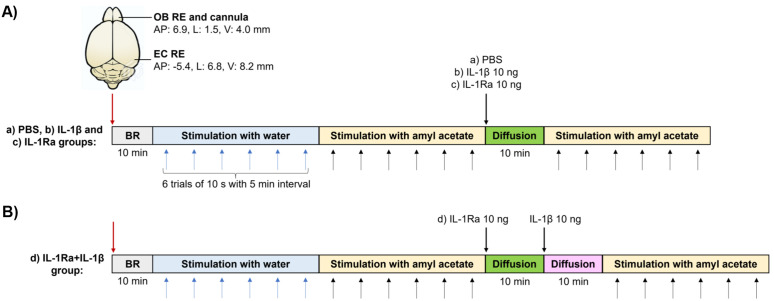
Experimental design to evaluate IL-1β effect on the electrical activity of the olfactory system. **A)** Recording electrodes (RE) were placed in the olfactory bulb (OB) and entorhinal cortex (EC), and a guide cannula was attached to the OB RE. Baseline LFPs were recorded for 10 min (BR, gray bar). Then, LFPs were recorded under olfactory stimulation, first with water (blue bar) as a negative control and next with amyl acetate (yellow bar). Six consecutive trials of 10 s with an interval of 5 min were performed. Afterwards, the following treatments were microinjected in the OB: **a)** PBS, **b)** IL-1β, or **c)** IL-1Ra (n = 6 per group). Treatments were allowed to diffuse in the OB (green bar), and a second round of olfactory stimulation with amyl acetate was executed. **B)** For the IL-1Ra + IL-1β group (d, n = 6), IL-1Ra was first microinjected in the OB and allowed to diffuse for 10 min (green bar), next IL-1β was administered and allowed to diffuse for another 10 min (pink bar). AP: anteroposterior, L: lateral, and V: vertical.

Recording of LFPs was carried out in an isolated, odor-free room. All the experiments were performed under ketamine/xylazine anesthesia (90/10 mg/kg) 30 minutes after stereotaxic surgery to allow the stabilization of the animals. Rats were connected to an amplifier (BE Light, EBNeuro, Italia) coupled to the Galileo NT version 3.0 software. LFPs were obtained by amplifying the signal at x1000 and fixed between 0.3 and 70 Hz according to reported parameters [24]. LFPs were recorded for 10 min during the rest period, and subsequently during olfactory stimulation. Rats were exposed to the odorant six times for 10 s with an interval of 5 min, first using water (odorless control) and then amyl acetate. Next, rats were divided into four groups (n = 6 per group): a) control microinjected with 0.4 μl of PBS, b) IL-1β 10 ng/0.2 μl, c) IL-1Ra 10 ng/0.4 μl, and d) IL-1Ra + IL-1β microinjected first with 10 ng of IL-1Ra/0.4 μl and 10 min later with 10 ng of IL-1β/0.2 μl. Microinjections (coordinates 6.9 mm anteroposterior, 1.5 mm lateral, and 4.0 mm vertical) were made with a stainless steel cannula (30 gauge, 50 µm internal diameter) connected to a 10 μl Hamilton microsyringe at a rate of 0.1 µl/min. Treatments were allowed to diffuse into the brain parenchyma for 10 min, and the olfactory stimulus with amyl acetate was presented as mentioned before (Fig 1).

### Local field potential analysis

LFPs were sampled at a frequency of 256 Hz, filtering the signal from 0.3 to 70 Hz. LFPs analysis was performed by taking a sample of 30 seconds from the beginning, middle, and end of the basal and post-treatment recordings. An automated method of the fast Fourier transform using the Galileo NT version 3.0 software (EBNeuro, Italy) and MATLAB software (MathWorks Inc. EE. UU.) was used to calculate the total spectral power, total signal frequency, and relative power of delta, theta, alpha, beta, and gamma oscillations.

### Olfactory evoked potential analysis

Evoked potentials are polyphasic signals generated in response to neuronal activity during sensory stimulation. Olfactory evoked potentials (OEPs) represent neuronal activation in the olfactory system when an odorant is detected by the OSN. We determined the OEPs generated in the OB. OEPs consist of a series of positive and negative components that produce a characteristic waveform. The components of OEPs display a distinctive latency (time interval between the stimulus onset and the peak of the component) and amplitude (distance [voltage] from the baseline to the peak of the component). Different reproducible positive (P) and negative (N) components are described for olfactory responses, which occur at about 100 ms (P1, N1), 200 ms (P2, N2), or 300 ms (P3, N3) after the stimulus [25,26]. We determined the amplitude and latency of the components P1, N1, P2, and N2.

Olfactory stimulation was performed in an isolated, odor-free room. Recordings were carried out in anesthetized rats (ketamine/xylazine, 90/10 mg/kg) placed in a stereotaxic frame to fix the head. After 10 min of IL-1β or IL-1Ra administration or both, the olfactory stimuli were presented in a cotton swab impregnated with 100 μl of water or amyl acetate (Sigma-Aldrich W504009) placed at 5 mm from the nostrils for 10 s, followed by a resting period of 5 min. This procedure was repeated another five times to complete six odorant expositions (Fig 1). During resting periods, the cotton swab was kept in a clean, closed vial. The moment at which each olfactory stimulus was made was labeled on the corresponding recording.

For subsequent OEPs analysis, amyl acetate trials were identified in recordings amplified at 200 ms per page. The amplitude of the OEPs components was determined by measuring the mean signal voltage with reference to baseline every 10 ms (the minimum sensitivity of the BE Light amplifier) until the corresponding peaks (maximum value) of the components P1, N1, P2, and N2 were identified in order of appearance. This procedure was performed for the 6 amyl acetate trials per rat to obtain the mean value per rat. The latency of the components (ms) was measured from the stimulus onset to the peak of each component for the 6 amyl acetate exposures per rat to obtain the mean value per rat [27].

### Histological analysis

At the end of the experiments, rats were euthanized and perfused with 0.9% saline solution followed by 4% paraformaldehyde. Brains were dissected and stored in the same fixative solution. Brain slices were obtained in serial coronal sections of 30 μm thickness at −20 °C with a cryostat (LEICA, CM1850) and directly mounted on gelatin-coated slides. Cresyl violet staining was performed to visualize electrode insertion in the OB and EC. Images were visualized using light microscopy.

### Il1r1 expression analysis in single-cell and bulk RNA-seq datasets

To determine the expression of the IL-1 receptor type 1 (Il1r1) in the whole rodent OB and at the single-cell level, RNA-sequencing datasets were downloaded from the Bioproject and Gene Expression Omibus (GEO) databases (NCBI). FASTQ files corresponding to bulk RNA-seq of the rat cerebellum, cortex, hippocampus, striatum, and OB in normal conditions (n = 3) were obtained (Bioproject accession: PRJEB39130) and aligned to the rat reference genome using the program HISAT2 [28]. The resulting files from the alignment were used to build read count matrices employing the R package Rsubread [29]. Differential gene expression was analyzed in R by using the package DESeq2 [30]. For single-cell expression analysis, raw sequence reads corresponding to single-cell RNA-seq of 18,048 cells from the mouse OB (n = 2) were downloaded (GEO accession: GSE121891). Raw reads were processed in the R package Seurat [31], where log normalization and scaling were applied. Principal component analysis and Louvain clustering were performed by using the Seurat functions RunPCA and FindClusters, respectively, while dimensionality reduction was performed by using the function RunUMAP; clusters were annotated employing the packages Celldex and SingleR [32]. All plots were completed in ggplot2 using R version 4.1.2.

### Statistical analysis

LFPs and OEPs data were analyzed with the Shapiro-Wilk test to determine distribution type. Total spectral power and total signal frequency in the OB and EC were analyzed with repeated measures two-way ANOVA and Tukey’s multiple comparisons test. The relative power of delta, theta, alpha, beta, and gamma oscillations, OEPs amplitude, and OEPs latency were analyzed with one-way ANOVA and Tukey’s multiple comparisons test. Statistical analysis was performed with GraphPad Prism version 10.2.

## Results

### Il1r1 is expressed in the rat and mouse OB

**Fig 2:**
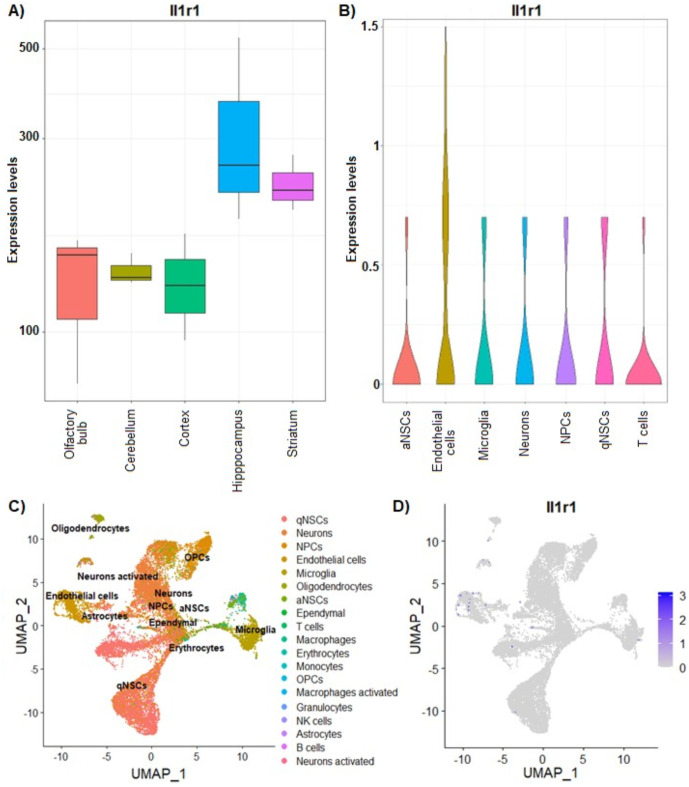
Il1r1 is constitutively expressed in the OB of rodents. **A)** Bulk RNA-seq expression analysis of Il1r1 in the rat OB, cerebellum, cortex, hippocampus, and striatum. The highest levels were detected in the hippocampus (p = 0.002869) and the striatum (p = 0.034318) compared to all other groups. **B)** Violin plot depicting the expression levels of Il1r1 detected by single-cell RNA-seq analysis in the cellular types of mice OB. Endothelial cells showed the highest expression levels (p = 0.0317) compared to all other groups. **C)** Uniform Manifold Approximation and Projection (UMAP) plot visualization of the specific cell types identified by single-cell RNA-seq analysis in the OB of mice. **D)** UMAP plot visualization of Il1r1 expression in OB cells from mice.

To determine whether microinjected IL-1β into the OB could activate its canonical receptor IL-1R1 in this brain area, we investigated the expression of this receptor using NCBI databases of both rat and mouse OB (Bioproject accession: PRJEB39130; GEO accession: GSE121891). Bulk RNA-seq analysis in the rat brain revealed that Il1r1 is constitutively expressed in several regions, including the OB; the highest levels were detected in the hippocampus (p = 0.002869) and the striatum (p = 0.034318) (Fig 2A). Meanwhile, single-cell RNA-seq analysis of 18,048 cells obtained from the mice OB showed a wide heterogeneity of cell types, where Il1r1 expression was specifically detected in activated neural stem cells (aNSCs), endothelial cells, microglia, neurons, neuronal progenitor cells (NPCs), quiescent neural stem cells (qNSCs), and T cells; being endothelial cells the group with the highest number of Il1r1-positive cells and the highest levels of Il1r1 expression (p = 0.0317) (Fig 2B-2D).

### IL-1β affects spectral power and signal frequency in the OB

**Fig 3:**
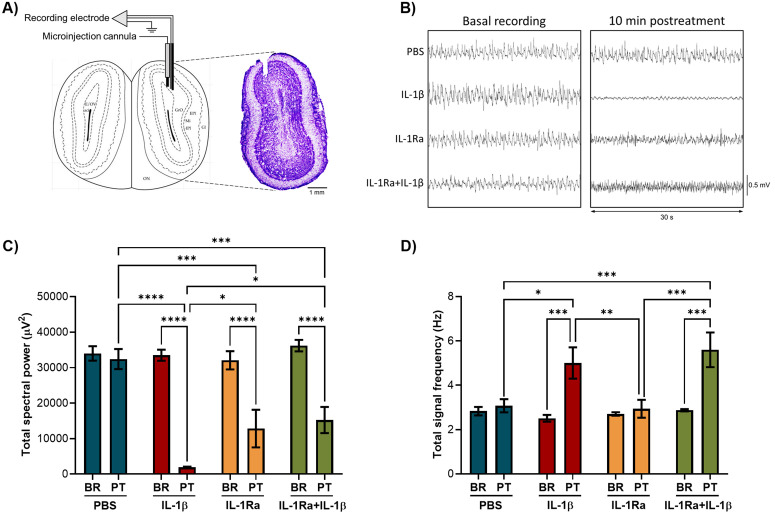
IL-1β microinjection in the OB decreased total spectral power and increased signal frequency. **A)** Representative histological confirmation of the recording electrode and microinjection site in the granule cell layer of the OB. **B)** Representative LFPs recordings in the OB before (basal) and after 10 min of PBS, IL-1β, IL-1Ra, and IL-1Ra + IL-1β microinjection. **C)** OB total spectral power and **D)** OB total signal frequency in the four groups (n = 6) before (BR) and after 10 min of treatments (PT). Two-way RM ANOVA followed by Tukey’s multiple comparisons test; *p < 0.05, **p < 0.01, ***p < 0.001, ****p < 0.0001.

The effect of IL-1β on the electrophysiological activity of the olfactory system was addressed by microinjecting IL-1β and its antagonist IL-1Ra into the rat OB and recording LFPs in the OB and EC. Recording electrode and microinjection site in the granule cell layer, which is the largest layer and the main source of LFPs in the OB [33], was confirmed by cresyl violet staining (Fig 3A). Total spectral power and total signal frequency of the LFPs in the OB before treatments (basal) were similar in all groups (Fig 3B-3D). After 10 minutes of IL-1β microinjection, total spectral power in the OB significantly decreased when compared to the basal activity, vehicle (PBS), IL-1Ra, and IL-Ra + IL-1β groups (Two-way RM ANOVA, F(3, 20)=11.83, p = 0.0001). Interestingly, IL-1Ra microinjection also decreased total spectral power, but its effect was lower than that of IL-1β (Fig 3C). Conversely, IL-1β significantly increased total signal frequency compared to PBS and IL-1Ra (Two-way RM ANOVA, F(3, 20)=5.357, p = 0.0072) (Fig 3D). Previous administration of IL-1Ra in the IL-1Ra + IL-1β group partially reverted the decrease in spectral power induced by IL-1β (p = 0.0106), but did not reverse the effect of IL-1β on signal frequency. These results suggest that IL-1β affects the electrophysiological activity in the OB.

### IL-1β increased gamma power in the OB

**Fig 4:**
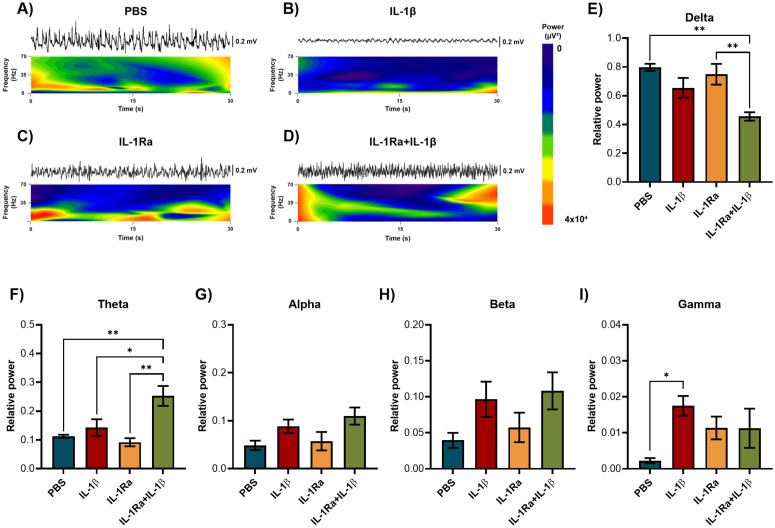
IL-1β increased the relative power of gamma oscillations in the OB. Representative LFPs spectrograms of the OB after treatment with **A)** PBS, **B)** IL-1β, **C)** IL-1Ra, and **D)** IL-1Ra + IL-1β. Relative power of E) delta (0−4 Hz), F) theta (4−8 Hz), G) alpha (8−12 Hz), H) beta (12−30 Hz) and I) gamma (30−70 Hz) oscillations in the OB after PBS, IL-1β, IL-1Ra and IL-1Ra + IL-1β microinjection (n = 6). One-way ANOVA followed by Tukey’s multiple comparisons test; *p < 0.05, **p < 0.01.

The total spectral power was decomposed to determine the relative power of delta, theta, alpha, beta, and gamma oscillations after treatments. IL-1β microinjection in the OB increased the relative power of gamma oscillations (30−70 Hz) compared to the control group (One-way ANOVA, F(3, 20)=3.312, p = 0.0410). Alpha (8−12 Hz) and beta (12−30 Hz) rhythms showed a tendency to increase in IL-1β and IL-1Ra + IL-1β groups (alpha: p = 0.0417, beta: p = 0.1041). Additionally, the IL-1Ra + IL-1β group displayed altered relative power of slow wave rhythms by decreasing delta oscillations (0−4 Hz) compared to PBS group (One-way ANOVA, F(3, 19)=6.942, p = 0.0024), and increasing theta rhythm (4−8 Hz) compared to PBS and IL-1Ra groups (One-way ANOVA, F(3, 19)=8.501, p = 0.0009) (Fig 4).

### IL-1β impaired the amplitude and latency of the OEPs induced by amyl acetate in the OB

**Fig 5:**
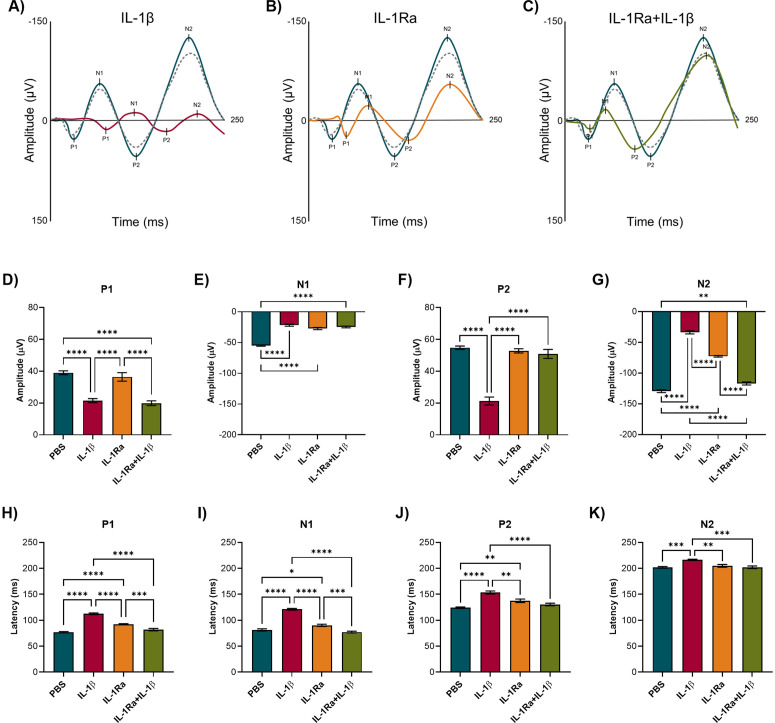
IL-1β impairs the olfactory response in the OB during olfactory stimulation. A-C) Representative OEPs in the experimental groups. The dotted line curve represents the olfactory basal response to amyl acetate before treatments; blue, red, orange, and green curves represent the olfactory response to amyl acetate after PBS, IL-1β, IL-1Ra, and IL-1Ra + IL-1β microinjection, respectively. D-G) Amplitude and H-K) latency of the components P1, N1, P2, and N2 in the PBS, IL-1β, IL-1Ra, and IL-1Ra + IL-1β groups (n = 6). One-way ANOVA followed by Tukey’s multiple comparisons test; *p < 0.05, **p < 0.01, ***p < 0.001 and ****p < 0.0001.

The effect of IL-1β microinjection in the OEPs produced in the OB by stimulation with amyl acetate was addressed by measuring the amplitude and latency of the components P1, N1, P2, and N2. Remarkably, IL-1β significantly reduced the amplitude of P1, P2 and N2 (One-way ANOVA, F(3, 20)=31.00, p < 0.0001; F(3, 20)=113.3, p < 0.0001; and F(3, 20)=396.0, p < 0.0001, respectively), and increased the latency of all components compared to PBS and IL-1Ra groups (P1: F(3, 20)=96.37, p < 0.0001; N1: F(3, 20)=106.6, p < 0.0001; P2: F(3, 20)=23.43, p < 0.0001; N2: F(3, 20)=11.18, p = 0.0002). Moreover, IL-1Ra pretreatment in the IL-1Ra + IL-1β group restored latency for all components and amplitude of P2 and N2. Nevertheless, the sole microinjection of IL-1Ra decreased N1 and N2 amplitude and increased P1, N1, and P2 latency, although in a significantly lower magnitude than IL-1β (Fig 5). These findings suggest that both IL-1β and IL-1Ra affect the olfactory response in the OB by decreasing the amplitude and increasing the latency of the OEPs. The effect of IL-1β on gamma oscillations during olfactory stimulation was also evaluated; however, gamma power did not differ in all the groups when they were exposed to amyl acetate (S2 Fig).

### IL-1β increased total signal frequency in the EC without modifying total spectral power

**Fig 6:**
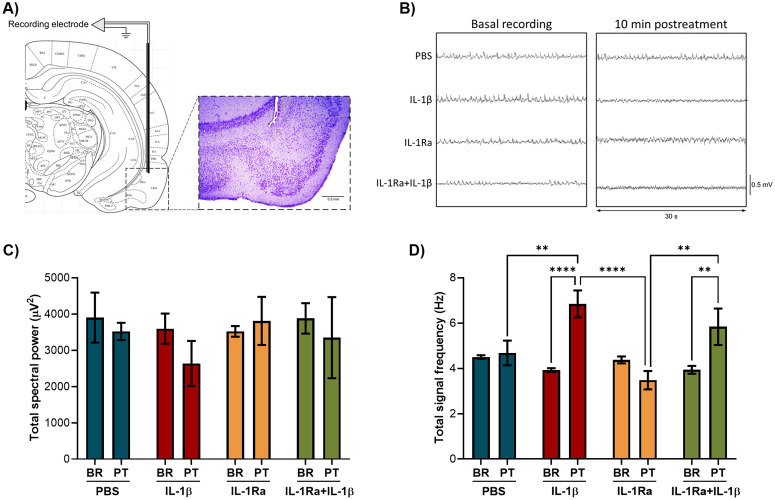
IL-1β microinjection in the OB increased total signal frequency in the EC without modifying total spectral power. **A)** Representative histological confirmation of the site of the recording electrode in the EC. **B)** Representative LFPs recordings in the EC before (basal) and after 10 minutes of PBS, IL-1β, IL-1Ra, and IL-1Ra + IL-1β microinjection (n = 6). **C)** EC total spectral power and **D)** EC total signal frequency in the groups before (BR) and after 10 min of treatment (PT). Two-way RM ANOVA followed by Tukey’s multiple comparisons test; **p < 0.01 and ****p < 0.0001.

To investigate whether the effect of IL-1β in the OB could be spread to other olfactory areas, LFPs were analyzed in the EC. In this case, neither IL-1β nor 1L-1Ra modified the total spectral power (Two-way RM ANOVA, F(3, 20)=0.4025, p = 0.7528). On the other hand, IL-1β significantly increased total signal frequency in the EC. This effect was not reversed by IL-1Ra pretreatment (Two-way RM ANOVA, F(3, 20)=8.362, p = 0.0008) (Fig 6).

### Rhythms in the EC displayed the same pattern as the OB

**Fig 7:**
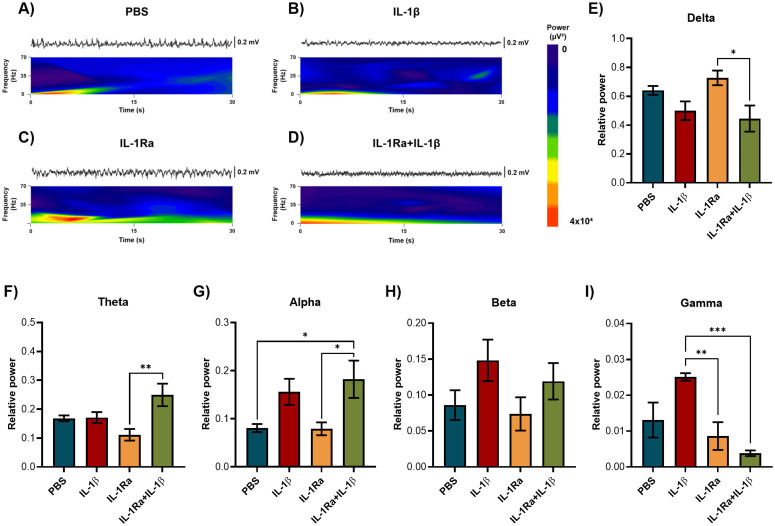
Rhythms in the EC displayed the same pattern as the OB. Representative LFPs spectrograms of the EC after treatment with **A)** PBS, **B)** IL-1β, **C)** IL-1Ra, and **D)** IL-1Ra + IL-1β. Relative power of E) delta (0−4 Hz), F) theta (4−8 Hz), G) alpha (8−12 Hz), H) beta (12−30 Hz) and I) gamma (30−70 Hz) oscillations in the EC after PBS, IL-1β, IL-1Ra and IL-1Ra + IL-1β microinjection (n = 6). One-way ANOVA followed by Tukey’s multiple comparisons test; *p < 0.05, **p < 0.01, and ***p < 0.001.

The relative power of the different rhythms in the EC showed a similar pattern to that observed in the OB. Delta and theta relative power decreased and increased in the IL-1Ra + IL-1β group, respectively (One-way ANOVA, F(3, 20)=4.183, p = 0.0188; F(3, 20)=5.388, p = 0.007). In addition, gamma oscillations in the EC increased in the IL-1β group (F(3, 20)=9.681, p = 0.0004), while alpha and beta rhythms showed a tendency to an increase (Fig 7), suggesting that IL-1β may modulate olfactory spreading from OB to secondary olfactory areas.

## Discussion

This study evaluated the effect of IL-1β and IL-1Ra on the electrical activity of the OB and EC. IL-1β and IL-1Ra microinjection in the OB significantly decreased total spectral power in the OB, while only IL-1β increased total signal frequency in the OB and EC. Moreover, IL-1β increased the OB and EC relative gamma power and affected the latency and amplitude of the OEPs in the OB. Notably, the sole administration of IL-1Ra also modified the evoked response in the OB. These results suggest a role for IL-1β and IL-1Ra in olfactory processing, which could be mediated by their binding to IL-1R1, since the expression of this receptor was identified in the rat and mouse OB. However, the effect of IL-1Ra on the electrical activity of the OB could also suggest an IL-1R1-independent mechanism.

The biological effects of IL-1β are mediated by its binding to IL-1R1, which is mainly present as a transmembrane protein rather than in its soluble form. When IL-1β interacts with IL-1R1, and recruits the accessory receptor IL-1RAcP to form a heterodimer that provides an anchor site for the myeloid differentiation primary response protein 88 (Myd88) and the subsequent activation of the transcription factor NF-κB. Soluble IL-1R1, the decoy receptor IL-1R2, and the endogenous antagonist IL-1Ra modulate IL-1β signaling. IL-1R1 is expressed in the CNS, mainly in vascular endothelial cells, but also at low levels in astrocytes, neurons, and microglia of brain areas such as the hypothalamus, pituitary, hippocampus, and cerebellum [34]. The physiological function of IL-1β in the brain is related to the regulation of body temperature, fever, sleep, and hippocampal long-term potentiation. In pathological conditions, astrocytes produce IL-1β to induce the release of more IL-1β and other proinflammatory cytokines, which promote neuroinflammation [12].

Few works have investigated the expression of IL-1β and IL-1R1 in the olfactory system under physiological conditions. The study of the expression of IL-1β in the development of the rodent OB displayed IL-1β immunoreactivity in all the layers of the OB from juvenile rats (P30) [35]. In adult rats, IL-1β was found in the granule and glomerular layers and in the olfactory tubercle [36,37]. Moreover, IL-1R1 mRNA was identified in the anterior olfactory nucleus by in situ hybridization [38]. Here, IL-1R1 expression was demonstrated by RNA-seq analysis in the rat and mouse OB, mainly in endothelial cells, but also at detectable levels in NSCs, microglia, and mature neurons, suggesting that IL-1β signaling may have a physiological function in the OB.

IL-1β increases blood-brain barrier (BBB) permeability in different CNS pathologies. This effect can be mediated by its interaction with IL-1R1 on the surface of the brain endothelial cells (BECs), as demonstrated by *in vitro* studies. The treatment of BECs with IL-1β increased their permeability to fluorescein and dextran, and MMP-9-induced degradation of the tight junction proteins ZO-1 and occludin [39,40]. Furthermore, leakage of brain endothelium induced by IL-1β could be triggered acutely, since BECs displayed a significant decrease of transendothelial electrical resistance and ZO-1 phosphorylation after 90 min of IL-1β treatment [41]. These findings suggest that IL-1β/IL-1R1 signaling in BECs could alter BBB permeability, which in turn could change the interstitial composition of solutes in the brain parenchyma and the electrical response of the OB.

On the other hand, systemic administration of LPS in rats increased the levels of IL-1β, TNF-α, IL-6, and inducible nitric oxide synthase (iNOS) in the OB [42]. In endothelial cells, nitric oxide (NO) induces the rearrangement of tight junction proteins and BBB opening in a mechanism dependent on guanylyl cyclase activation. Furthermore, the activation of NMDA receptors induces the NO synthesis in endothelial cells; in turn, the produced NO can reach astrocytes and neurons and potentiate glutamate release [43]. This information suggests that under inflammation, NO synthesis in OB endothelial cells may alter BBB permeability and glutamate release.

IL-1β microinjection in the OB depressed the electrical activity by reducing total spectral power; however, IL-1Ra also altered the total spectral power in the OB. Notably, the effect of IL-1β on the electrical activity of the OB occurred only 10 minutes after its microinjection, suggesting that IL-1β may modify neurotransmission. In this context, it was reported that IL-1β can modulate ion currents of sodium, calcium, and potassium through voltage-gated channels within seconds to hours [44]. Previous studies demonstrated that IL-1β increased the levels of serotonin, dopamine, and noradrenaline when administered in the hypothalamus, and decreased acetylcholine levels in the hippocampus. Besides, IL-1β enhanced synaptic inhibition in the hippocampus presumably by increasing inward permeability of chloride through GABA receptors [19,22]. In awake rats, IL-1β decreased discharge rate in the preoptic area of the hypothalamus and the magnocellular basal forebrain neurons [45]. Another study proposed that microglial IL-1β production after TLR4 activation can impair GABA receptor activity and GABA synthesis [46]. Additionally, IL-1β increased calcium influx into neurons through NMDA receptors, promoting hyperexcitability in both *in vitro* and *in vivo* studies [44]; meanwhile, Anakinra (recombinant IL-1Ra) controlled seizures in refractory epilepsy and a rodent model of status epilepticus [47]. All this information suggests that IL-1β can modify inhibitory and excitatory neurotransmission.

Sensory signals in the OB arrive via OSN axons, which make contact in the glomerulus with dendrites of mitral and tufted cells, whose neuronal bodies are located in the mitral cell layer and external plexiform layer, respectively. Secondary dendrites of MTC form synapses with granule cells that extend their dendrites from the granule cell layer to the external plexiform layer [48]. The granule cell layer is the largest layer of the OB and is the main source of LFPs in this olfactory area. MTC firing rate is mainly regulated by GABAergic granule cells but also by glomerular interneurons; therefore, LFPs representing MTC firing patterns match those of the granule cells, since they are closely connected [33]. The relationship between MTC and granule cells suggests that IL-1β and IL-1Ra effects in the LFPs originated in the granule cell layer may represent the effects of these cytokines in MTC LFPs.

IL-1β microinjection in the OB altered fast oscillations by increasing gamma frequency in the OB and EC; remarkably, IL-1Ra pretreatment seems to revert this effect. Gamma rhythm is frequently present in sensory systems. The first description of gamma oscillations in the OB was made by Adrian in 1950, who measured LFPs in anesthetized cats, rabbits, and hedgehogs. In 1990, Eeckman and Freeman proposed that frequency and power of gamma oscillations represent a measure of the probability of MTC activation; later studies supported the role of gamma oscillations in olfactory processing [49]. In the OB, gamma oscillations are prominent in awake animals and are present during rhythmic respiration even in the absence of odor stimulation. Gamma oscillations in the OB have a key role in appropriate odor discrimination, which is a process associated with lateral inhibition between mitral (glutamatergic) and granule (GABAergic) cells. Pharmacological inhibition of GABA_A_ receptors enhances low gamma (40−70 Hz) synchronization and impairs odor discrimination, suggesting that gamma oversynchronization impairs olfactory function [24]. Notably, increased low gamma oscillations were associated with altered synaptic transmission in AD mouse models (APP/PS1 and 3xTg) [50]. In addition, gamma, delta, and theta rhythms increased in the OB of mice with traumatic brain injury under olfactory stimulation [51]. This information suggests that the increase of gamma oscillations induced by IL-1β in the OB and EC represents a sign of olfactory impairment. Nevertheless, it was also reported reduced gamma power in the OB of aged mice and a mouse model of tauopathy (P301S) [52,53].

Besides gamma, theta, and beta rhythms are important for olfactory processing. Particularly, gamma rhythms depend on brain state, differing in awake and anesthetized animals. For instance, under chloral hydrate and pentobarbital anesthesia, beta power in the OB increased while gamma power decreased, but with urethane anesthesia, both rhythms decreased [54]. Theta oscillations (4–8 Hz) are involved in long-term potentiation in the glomerular, external tufted, mitral, and granule cells, facilitating odor processing, discrimination, and perception [55]. Beta oscillations (15–35 Hz) in the OB are also observed during the respiratory cycle. Whereas odor-evoked gamma oscillations occur in the transition point between inhalation and exhalation, beta oscillations occur during early exhalation and can extend to the end of inhalation [56]. IL-1β microinjection tended to increase beta oscillations in the OB and EC, and theta rhythm only increased with the simultaneous treatment with IL-1Ra + IL-1β. Interestingly, delta power (0–4 Hz) was reduced only in the IL-1Ra + IL-1β group, suggesting the possible implication of a physiological role for IL-1β in the rhythms of slow waves in the OB and EC.

OEPs are polyphasic signals that consist of a series of positive and negative components with a characteristic latency and amplitude. The components P1, N1, and P2, N2 that emerge near 100 and 200 ms, respectively, are related to odor threshold and identification [25]. IL-1β microinjection in the OB reduced P1, P2, and N2 amplitude and increased the latency of all components. Interestingly, IL-1Ra pretreatment restored latency for all components and the amplitude of P2 and N2. Odorant-evoked responses may change with brain state. For instance, olfactory response in the anterior piriform cortex was lower under deep anesthesia than under a shallower stage of anesthesia; notably, olfactory response in the OB was high in deep and light anesthesia [57]. During anesthesia, MTCs are selectively activated by odors, but also display a respiratory pattern that modulates gamma and beta frequencies [58,59]. In anesthetized rats, odorant stimulation induces gamma and beta oscillations, which appear alternately with the respiratory cycle. The simultaneous recording of mitral cells activity and LFPs in freely breathing anesthetized rats showed that odorant stimulation induces spike trains that occur simultaneously with gamma oscillations to form odor-specific temporal patterns [60]. In this work, IL-1β increased gamma oscillations of the OB in resting conditions, but not during olfactory stimulation.

It is important to note that IL-1Ra alone also decreases the total spectral power in the OB and affects the olfactory evoked response to amyl acetate by decreasing N1 and N2 amplitudes and increasing P1, N1, and P2 latencies. Loscher et al. 2003 highlight that most studies that have examined the blocking effect of IL-1Ra on IL-1β function did not evaluate the effect of IL-1Ra alone. These authors studied the effect of IL-1β and IL-1Ra on LTP induced in the mouse and rat hippocampus, finding that the sole administration of IL-1Ra mimics the effects of IL-1β, namely reduction of glutamate release and LTP amplitude, and increase JNK activation, in an independent manner of IL-1R1 [61]. IL-1β production in the CNS was demonstrated in an early study, in which increased neuronal activity in the rat hippocampus during LTP induced IL-1β expression. Conversely, LTP amplitude decreased with IL-1Ra treatment, possibly by blocking IL-1R1, although the interaction between IL-1Ra and IL-1R1 was not determined [62]. In another study in mice, IL-1Ra was capable of reversing the secretion of several neurotransmitters from the hypothalamus induced by the granulocyte-macrophage colony-stimulating factor (GM-CSF). However, the sole administration of IL-1Ra decreased norepinephrine levels [63]. These findings, as well as the present results, suggest that IL-1Ra has additional effects beyond IL-1R1 antagonism.

Neuronal activity generates extracellular electrical fields, which can be recorded from the scalp in the electroencephalogram (EEG), by measuring magnetic fields in the skull (magnetoencephalography, MEG), by subdural electrodes on the surface of the cerebral cortex or electrocorticography (ECoG), or by measuring LFPs when the electrode is placed inside a specific brain area. EEG, MEG, and ECoG account for the electrical activity in the superficial layers of the cortex, while LFPs represent the electrical events at deeper brain locations [64]. Although evoked potentials are commonly recorded using surface electrodes (EEG), especially in cortical regions, LFPs have also been used to study stimulus-evoked activity in cortical and subcortical structures, including the olfactory bulb.

Several studies have successfully employed LFPs to characterize odor-evoked potentials in olfactory areas both in anesthetized and awake rodents. For instance, the spaciotemporal distribution of evoked responses in olfactory areas was investigated in anesthetized rats after electrical stimulation of the OB. LFPs were measured by placing recording electrodes in the OB, anterior piriform cortex (PC), posterior PC, and EC to determine the occurrence of early and late evoked potential components. The presence of both components was observed in the anterior and posterior PC and EC, while only the early component occurred in the OB [65]. LFPs were also recorded in the EC after electrical stimulation of the lateral olfactory tract to analyze the evoked potential elicited by the stimulus. Authors observed a response with three components (N1, P1, and N2) in awake and lightly anesthetized rats, which was suppressed in bulbectomized rats [66]. In another study, electrical stimulation of the OB in rats was used as a learning paradigm to discriminate the consumption of sucrose and quinine, as well as to associate the learning with the evoked field potentials measured by deep electrodes in the PC, EC, and dentate gyrus of the hippocampus [67].

Evoked potentials elicited by amyl acetate, phenylethyl alcohol, and eugenol were measured in the rat OB and scalp, by placing recording electrodes in the granular layer of the OB and subcutaneously in the skin over the nose, respectively. Therefore, LFPs were recorded in the OB, while EEG was recorded over the nose. Olfactory stimulation was performed using an olfactometer to test different conditions, including nasal cannula position, stimulus duration, flow rate, odorant concentration, and interstimulus interval. The latency of OEP components was measured from the stimulus onset to the maximum voltage of the component. The OEP component’s amplitude was the difference between the voltage at the peak of the component and the baseline. Three components were identified in the OB (P60, N90, and P140), with the highest amplitude when olfactory stimulation was made with 10% amyl acetate. This response was also observed when OEPs were recorded in the skin overlaying the nose, but amplitude was up to 25% of that observed in the deep OB OEPs, and with an opposite polarity. Furthermore, the amplitude of OEPs decreased when the nasal cannula was moved away from the nostril [68]. These early results are consistent with those found in the present study. Although we did not use an olfactometer for stimulation, the components P1, N1, and P2 showed a similar latency, albeit with a minor amplitude, likely because the odorant was administered away from the nose. Therefore, the measurement of LFPs in our study provides a reliable and established method to assess stimulus-evoked neural activity in the OB and EC.

## Conclusions

There is a large number of inflammatory molecules that modulate the olfactory system. However, the mechanisms that underlie their interaction remain mostly unexplored. We provide for the first time evidence demonstrating a modulatory effect of IL-1β in the electrophysiological function of the olfactory bulb, even when its receptor is mainly present in non-neuronal cells such as endothelial cells. Our results support a key role of inflammatory molecules in the correct functioning of olfaction, which requires further study to understand the complex interaction of these molecules with the cells that reside in olfactory brain structures in healthy and pathological conditions.

## Supporting information

S1 Fig Effect of 10 ng of IL-1β and IL-1Ra in a preliminary recording of the LFPs in the OB. A) A single dose of 10 ng of IL-1β decreased the amplitude of LFPs in the OB immediately after its microinjection, reaching a maximum effect at approximately 10 min and remaining constant for at least 45 min. B) Similar results were observed with a single dose of 10 ng of IL-1Ra. a) First 15 min of the LFPs recording representing 5 min of the basal activity before IL-1β or IL-1Ra microinjection into the OB, and 10 min after microinjection. b) LFPs recording of the 15–30 min and c) 30–45 min post microinjection, indicating the temporal point (every 5 min) in which the six trials with amyl acetate were performed.
(TIF)

S2 Fig Gamma power did not differ in the groups during olfactory stimulation. A) Gamma oscillations expressed as the mean relative power of the six trials to amyl acetate in each group. B) Gamma relative power in each trial with amyl acetate in the four groups. One-way ANOVA (A) and Two-way RM ANOVA (B) followed by Tukey’s multiple comparisons test, ns.
(TIF)

S1 File PBS R1 and R2 LFP recordings. Raw data of PBS R1 and R2 LFP recordings.
(ZIP)

S2 File PBS R3 and R4 LFP recordings. Raw data of PBS R3 and R4 LFP recordings.
(ZIP)

S3 File PBS R5 and R6 LFP recordings. Raw data of PBS R5 and R6 LFP recordings.
(ZIP)

S4 File IL-1b R1 and R2 LFP recordings. Raw data of IL-1b R1 and R2 LFP recordings.
(ZIP)

S5 File IL-1b R3 LFP recordings. Raw data of IL-1b R3 LFP recordings.
(ZIP)

S6 File IL-1b R4 LFP recordings. Raw data of IL-1b R4 LFP recordings.
(ZIP)

S7 File IL-1b R5 and R6 LFP recordings. Raw data of IL-1b R5 and R6 LFP recordings.
(ZIP)

S8 File IL-1RA R1 and R2 LFP recordings. Raw data of IL-1RA R1 and R2 LFP recordings.
(ZIP)

S9 File IL-1RA R3 and R4 LFP recordings. Raw data of IL-1RA R3 and R4 LFP recordings.
(ZIP)

S10 File IL-1RA R5 and R6 LFP recordings. Raw data of IL-1RA R5 and R6 LFP recordings.
(ZIP)

S11 File IL-1b_IL-1RA R1 and R2 LFP recordings. Raw data of IL-1b_IL-1RA R1 and R2 LFP recordings.
(ZIP)

S12 File IL-1b_IL-1RA R3 and R4 LFP recordings. Raw data of IL-1b_IL-1RA R3 and R4 LFP recordings.
(ZIP)

S13 File IL-1b_IL-1RA R5 and R6 LFP recordings. Raw data of IL-1b_IL-1RA R5 and R6 LFP recordings.
(ZIP)

S14 File Processed data. Processed data of LFP recordings.
(ZIP)
