# Supplementary figures and images for: Interleukin-1β depresses neuronal activity in the rat olfactory bulb even during odor stimulation
Source: PLoS One. 2025 Sep 18;20(9):e0332592. doi: 10.1371/journal.pone.0332592 (PMC12445527; doi:10.1371/journal.pone.0332592)

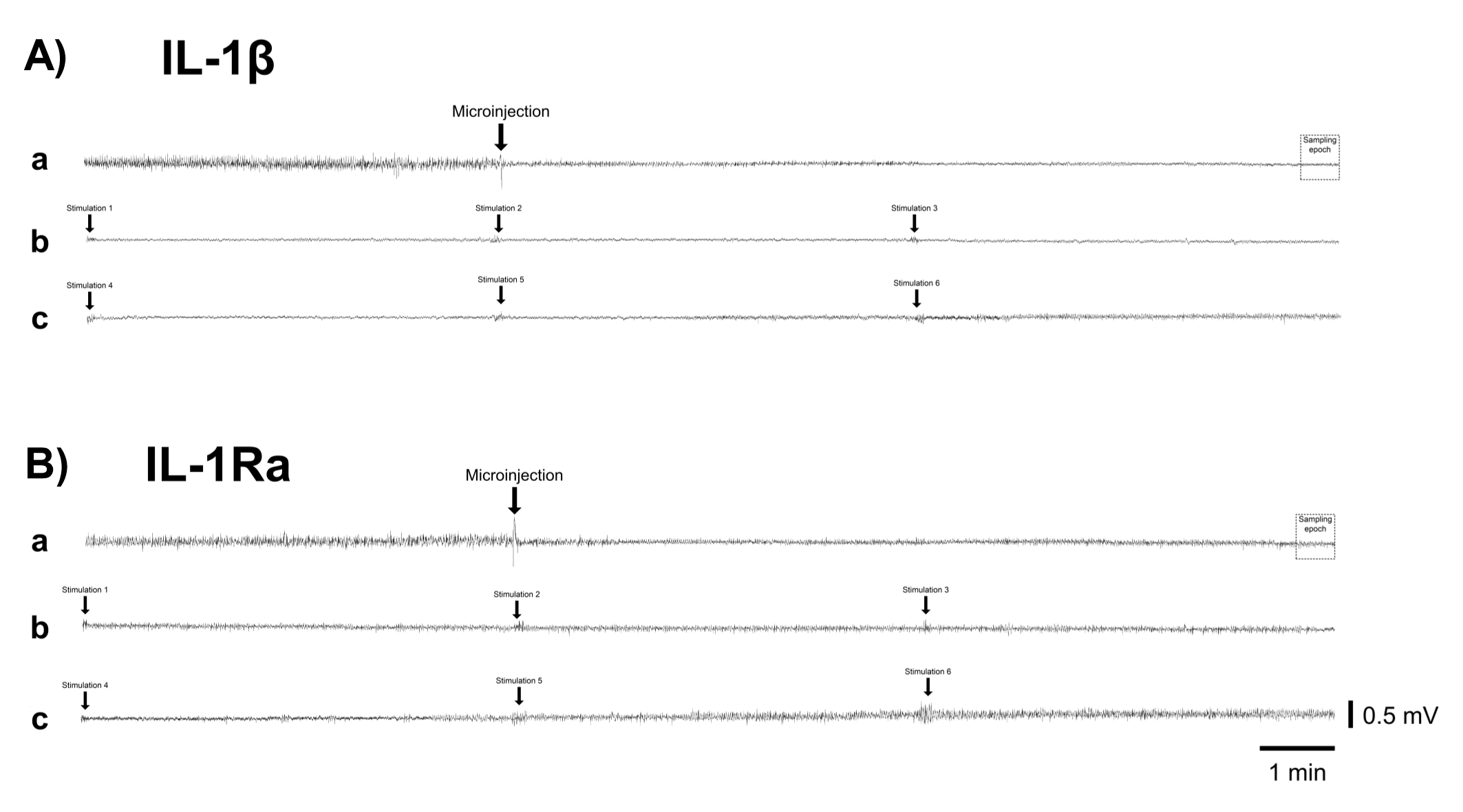

Supplement: S1 Fig — A) A single dose of 10 ng of IL-1β decreased the amplitude of LFPs in the OB immediately after its microinjection, reaching a maximum effect at approximately 10 min and remaining constant for at least 45 min. B) Similar results were observed with a single dose of 10 ng of IL-1Ra. a) First 15 min of the LFPs recording representing 5 min of the basal activity before IL-1β or IL-1Ra microinjection into the OB, and 10 min after microinjection. b) LFPs recording of the 15–30 min and c) 30–45 min post microinjection, indicating the temporal point (every 5 min) in which the six trials with amyl acetate were performed. (TIF) [file pone.0332592.s001.tif]

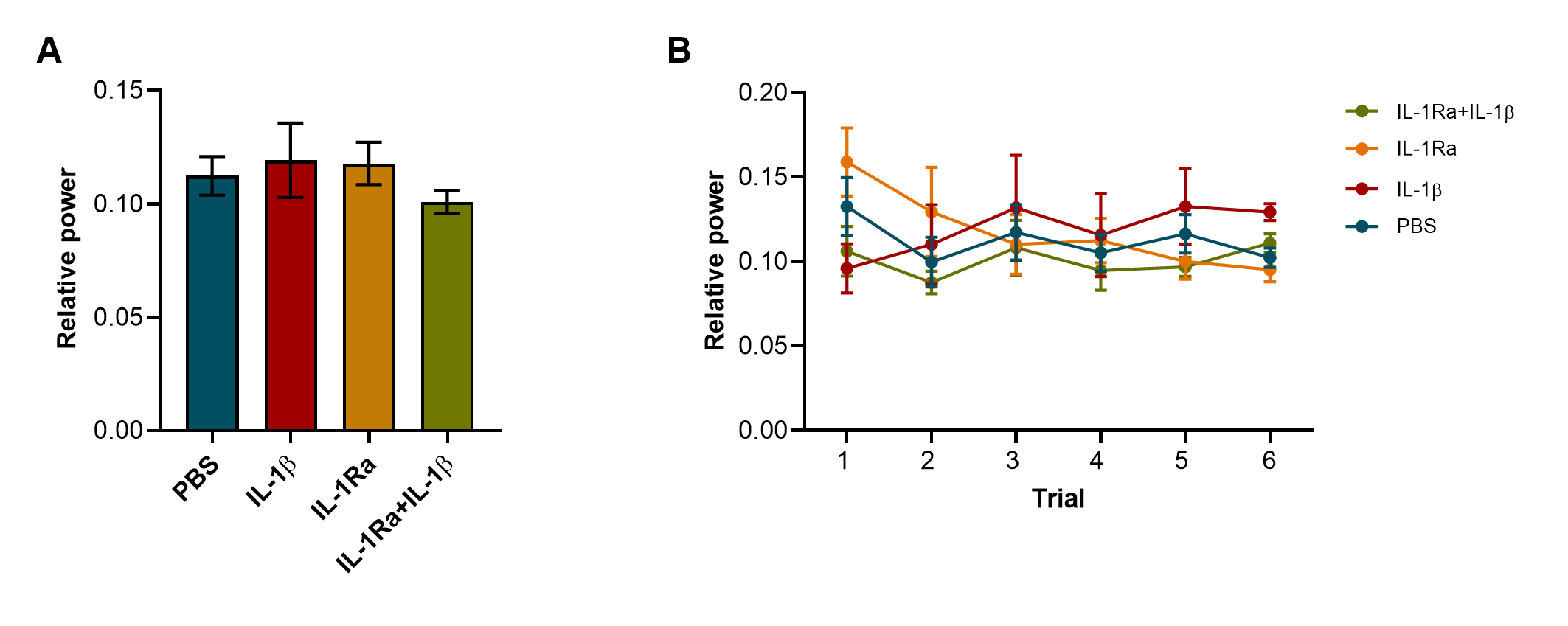

Supplement: S2 Fig — A) Gamma oscillations expressed as the mean relative power of the six trials to amyl acetate in each group. B) Gamma relative power in each trial with amyl acetate in the four groups. One-way ANOVA (A) and Two-way RM ANOVA (B) followed by Tukey’s multiple comparisons test, ns. (TIF) [file pone.0332592.s002.tif]
